# Health, Transport and the Environment: The Impacts of the COVID-19 Lockdown on Air Pollution

**DOI:** 10.3389/fpubh.2021.637540

**Published:** 2021-04-13

**Authors:** Luigi De Maria, Antonio Caputi, Silvio Tafuri, Enza Sabrina Silvana Cannone, Stefania Sponselli, Maria Celeste Delfino, Antonella Pipoli, Vito Bruno, Lorenzo Angiuli, Nicola Mucci, Caterina Ledda, Luigi Vimercati

**Affiliations:** ^1^Interdisciplinary Department of Medicine, University of Bari, Bari, Italy; ^2^Unit of Occupational Medicine, University Hospital of Bari, Bari, Italy; ^3^Department of Biomedical Science and Human Oncology, University of Bari, Bari, Italy; ^4^Regional Agency for Environmental Protection Agenzia Regionale per la Protezione Ambientale (ARPA) Puglia, Bari, Italy; ^5^Department of Experimental and Clinical Medicine, University of Florence, Florence, Italy; ^6^Occupational Medicine, Department of Clinical and Experimental Medicine, University of Catania, Catania, Italy

**Keywords:** SARS-CoV-2, COVID-19 lockdown, air pollution, transport, environment

## Abstract

Lockdown measures were initiated in Italy on March 9th after the start of the SARS-CoV-2 epidemic to flatten the epidemic curve. The aim of the present study was to assess the impact of restrictive measures in the Apulia Region, southern Italy, on air quality from March to April 2020. We applied a dual-track approach. We assessed citizen mobility and vehicle traffic with mobility network data and information obtained from satellite tracking, and we evaluated and compared pollutant concentration data as measured by monitoring stations maintained by the Regional Agency for Environmental Protection and Prevention of Apulia (ARPA). The results showed a decrease in the weekly mean NO_2_ concentration recorded by urban traffic stations during the lockdown period. In particular, in the city of Bari, the average NO_2_ concentration decreased from 62.2 μg/m^3^ in March 2019 to 48.2 μg/m^3^ in March 2020. Regarding PM10 levels, the average concentrations at the individual traffic stations showed no particular variation compared to those in the same months of the previous year, except for Bari-Caldarola Station in March 2019/2020 (*p*-value < 0.001) and in April 2019/2020 (*p*-value = 0.04). In particular the average in March 2019 was ~26.9 μg/m^3^, while that in March 2020 was ~22.9 μg/m^3^. For April, the average concentration of PM10 in 2019 was 27.9 μg/m^3^, while in 2020, the average was ~22.4 μg/m^3^. This can be explained by the fact that PM10 levels are influenced by multiple variables such as weather and climate conditions and desert dust advections.

## Background

Air pollution has become a growing concern in the past few years because of its effects on public and individual health. Air pollutants, such as particulate matter (PM), ozone, nitrogen oxide, sulfur dioxide, volatile organic compounds, dioxins, polycyclic aromatic hydrocarbons, persistent organic pollutants, carbon monoxide, asbestos and heavy metals are considered risk factors for various diseases (1–12). Long-term exposure to air pollutants is also associated with a variety of cancers (13–17). PM is most closely associated with the increased incidence of lung cancer (18). According to the World Health Organization (WHO) 3 million deaths/year in 2016 were linked to exposure to outdoor air pollution (19).

The Coronavirus Disease 2019 (COVID-19) restriction measures have been mainly oriented on flattening the epidemic curve, but at the same time confinement of the population, reduction of public transport and economic activities let to a considerable decrease in road traffic, and consequently, in levels of urban air pollution.

Italy has been the first European country to be severely affected by COVID-19 (20–22). The first case of COVID-19 was detected on February 20, 2020 in Codogno, near Milan (23) and since then, behavioral, clinical and government interventions have been undertaken to contain the outbreak and prevent the collapse of public health systems (24). On February 23, 2020, the Italian Government declared the area of the outbreak “red zone,” limiting social, cultural, economic activities, transports and schools (25). The Decree of the President of the Council of Ministers (March 9, 2020) established that any movement of individuals throughout the national territory was to be avoided. These measures reduced the free movement of citizens, with a large impact on the transportation sector, one of the most affected^1^. By 25 March, everything not related to food provision, pharmacies, health services and basic functioning of the country (the so defined “non-essential activities”) had been shut down (26).

Recently, scientists from the Royal Netherlands Meteorological Institute (KNMI) tried to document the effects of the drastic reduction in vehicular traffic on air quality and pollution levels through the analysis of data from Copernicus Sentinel-5P satellite. Nitrogen dioxide (NO_2_) was used as the tracer, not only because it is an indicator of the mixture of all pollutants derived from vehicular traffic but also because it responds rapidly to emission variations. From 13 March to 13 April 2020, satellite images showed a decrease in the mean NO_2_ concentration across Europe compared to the same months in the previous year. In Italy, these effects were more significant in the Po valley area, although satellite images also showed an important decrease in the NO_2_ concentration in the rest of the national territory ^2^.

The aim of the present study was to assess the impact of the restrictive measures on air quality in the regional territory of Apulia, southern Italy, with a focus on NO_2_ and PM10 levels in the municipal area of Bari in the months of March and April 2020.

## Methods

We applied a dual-track approach with assessment of the following:

- Citizen mobility in Italy and monitoring of vehicle traffic during the “lockdown period” (March–May 2020) by comparing mobility network data and information resulting from satellite tracking. From January to April 2020, the Observatory on the Italian Public Accounts compared data provided by digital companies such as Google and Apple. Specifically, data related to driving directions that were available on the website “Mobility Trends Reports” by Apple were compared to Community Mobility Reports by Google, which show changes in visits to places such as grocery stores and parks. From 11 March to 3 May 2020, the National Observatory of Survey on Mobility Style and Behaviors of Italians (AUDIMOB) of the High Institute for Transport Education and Research (ISFORT) carried out a study on the mobility of Italian citizens in the age group of 14–80 years using telephone and online interviews. Data relating to vehicle traffic during the “lockdown period” are available from digital platforms such as the “Mobility Data Lab” and “Enel X City Analytics.” The “Mobility Data Lab” is a digital platform available to the community that collects data from millions of vehicles equipped with on-board telematic devices and is able to provide mileage data anonymously. “Enel X City Analytics” is a free mobility map by the Enel X network that, in full compliance with privacy protection standards, allows users to view several data in the selected geographical area: (the percent changes in journeys compared to those on the same day in the previous week and the pre-emergency reference period; the percent changes in average distances traveled compared to those on the same day in the previous week and pre-emergency reference period; input-output flows in the selected geographical area).
- Data resulting from the analysis of pollutant concentrations measured by monitoring stations maintained by the Regional Agency for Environmental Protection and Prevention of Apulia (ARPA) from March-April 2020. In this period ARPA carried out a study to monitor variations in pollutants following the main events that occurred during the lockdown period, such as school closures (Decree of March 4, 2020, issued by the President of the Council of Ministers) and the extension of restrictive travel measures to the whole national territory (Decree of March 9, 2020, issued by the President of the Council of Ministers). For each provincial capital, a monitoring station that was exposed to vehicular traffic was selected. The six identified stations were classified as 4 urban traffic stations (BARI—c.so Cavour, BRINDISI—via dei Mille, LECCE—Piazza Libertini, TARANTO—via Alto Adige) and 2 urban background stations (FOGGIA—via Rosati, BARLETTA—via Casardi). Furthermore, into the city of Bari we considered the 5 monitoring stations in the metropolitan area: 3 traffic stations (Bari-Caldarola, Bari-Cavour, Bari CUS) and 2 urban background stations (Bari Carbonara, Bari Kennedy). The 3 traffic stations record the emissions concentrations of pollutants due to vehicular traffic, while the 2 urban background stations record pollutants concentrations which originate from multiple sources (industries, traffic, residential heating). Consequently, for the aim of our study we only evaluated the data from the 3 traffic stations. All the monitoring stations are part of the network of Air Quality Monitoring Stations (RRQA) managed by local environmental agency (ARPA Puglia). RRQA network consists of samplers/analyzers that detect and analyze the concentration of air pollutants (SO_2_, NOx, CO, O_3_, C6HS), and particulate matters continuously (24 h/day) all year round according to reference technical standards (UNI EN 14211:2012 for NO_2_ and UNI EN 16450:2017 for PM10) (27, 28). NOx air sampling takes place hourly with TELEDYNE Mod. T200 in Bari-Kennedy station and with TELEDYNE Mod. 200E in the remaining Bari stations. PM10 air sampling takes place daily with ENVIRONMENT Mod. MP101M in Bari-Carbonara station, SWAM FAI INSTRUMENTS Mod. 5A DUAL CHANNEL in Bari-Cavour and Bari-Caldarola stations, SWAM FAI INSTRUMENTS Mod. 5° in Bari-Kennedy and Bari-CUS stations. Quality Assurance/Quality Control (QA/QC) activities are regularly conducted by ARPA Puglia in accordance with Italian law (29) since 2013, at first with the QA/QC of the oxide analyzers of nitrogen (NOx) and ozone (O_3_) and afterwards adding the controls on the monoxide analyzers carbon (CO) and the verification of the sampling flows of the particulate analyzers/samplers atmospheric (PM10 and PM2.5).

Statistical analysis was performed with IBM SPSS Statistics Version 26. Kolomorgov-Smirnov and Shapiro-Wilk tests were performed to verify that the variables followed a Gaussian distribution and to choose appropriate statistical tests. It was found that not all variables distribute normally so Wilcoxon's non-parametric method was chosen to compare the means of the variables. *P*-values < 0.05 were considered statistically significant.

## Results

### Italian Mobility During the “Lockdown Period”

Data analysis showed that in Italy mobility almost ceased during the lockdown period. In particular, on 13 April, travel to the workplace decreased by more than 60%, and a reduction in the use of public transportation and cars occurred (90 and 85%, respectively) compared to use on 13 January 2020 ^3^.

AUDIMOB research showed a decrease in the mobility rate “in the strictest sense” (percentage of interview subjects who, during the day, traveled at least once by any mode of transportation, with the exception of walking <5 min) from 85% in 2019 to 32% in the lockdown period. Furthermore, there was a sharp decline in public and exchange mobility (public transportation, combination of modes) from 12.2 to 4.1% during the same period. In contrast, the study noted an increase in the proportion of active mobility from 25.1% in 2019 to 34.9% in 12 March−3 May ^4^.

### Vehicle Traffic During the “Lockdown Period”

“*Mobility Data Lab*” data showed that in Italy, the daily journey (number of vehicles for kilometers) decreased considerably starting in the second half of March, both for heavy vehicles (−38%) and for light vehicles (−71%). In particular, on 12 April 2020, it was recorded the highest reduction in the number of vehicles for kilometers compared to the same day in February (pre-lockdown) for both light vehicles (7,633,011 on 12 January 2020 vs. 430,477 on 12 April 2020; −94.4 %) and for heavy vehicles (2,213,498 of 12 January 2020 vs. 36,301 of 12 April 2020; −98.4%). According to national data, the lowest value in Apulia Region (39,237 vehicles for kilometers) was recorded on 12 April 2020 (approximately a month after the beginning of lockdown) ^5^.

“*Enel X City Analytics”* data showed that in the Apulia region, the percent changes in the numbers of journeys and kilometers traveled, compared to the standard reference period of 13 January−16 February, reached minimum values of −90 and −92% on 13 April 2020. On 12 April 2020, the maximum variation in input-output flows occurred (−94 and −93%, respectively).

### The Analysis of Pollutant Concentrations

The analysis of ARPA data showed that during the lockdown period, a decrease in the weekly mean NO_2_ concentration recorded by urban traffic stations occurred. In particular, the Bari – Corso Cavour station recorded a weekly mean NO_2_ concentration of 36.57 μg/m^3^ in the fourth week of lockdown (19 March 2020–25 March 2020). In the same week of 2015 this value was 94.71 μg/m^3^, in 2016 was 92.29 μg/m^3^ and in 2017 was 69.43 μg/m^3^. The same traffic station recorded a weekly mean NO_2_ concentration of 29.43 μg/m^3^ in the fifth week of lockdown (26 March 2020–01 April 2020). In the same week of 2015 this value was 96.43 μg/m^3^, in 2016 was 76.71 μg/m^3^ and in 2017 was 69.14 μg/m^3^.

At each monitoring station, data for each day were recorded during both the pre-emergency and lockdown periods. In all cases, a decrease in the mean NO_2_ concentration was evident, especially during peak hours.

Regarding particulate matter, the relationships between PM10 and PM2.5 concentration reductions and emergency restrictions were unclear. The particulate matter concentration is influenced by different factors, such as weather conditions (e.g., sand from desert areas). Indeed, during the last days of March, despite restrictive measures, the monitoring stations recorded levels exceeding the daily legal limit for PM10; this was presumably connected to the occurrence of desert dust from the Caspian Sea ^6^.

### Air Quality in the Municipal Area of Bari in 2019 vs. 2020

On the website http://www.arpa.puglia.it/web/guest/meta-aria, all environmental monitoring data resulting from the ARPA stations are available. An analysis of NO_2_ data collected during the lockdown period revealed variations in the average concentrations at the traffic stations in the months of March and April 2020 compared with those in the previous year.

**Figure 1:**
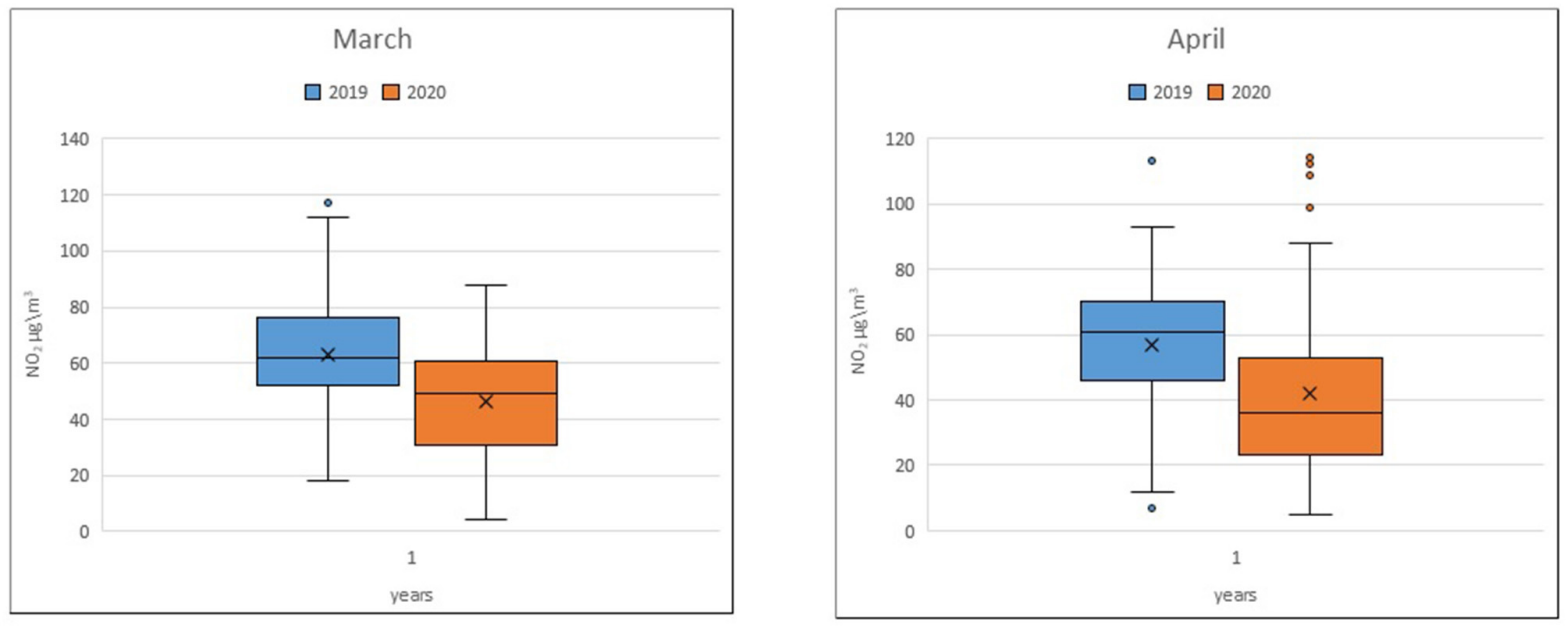
NO_2_ monthly average at the traffic stations.

The average in March 2019 was ~62.2 μg/m^3^, while that in March 2020 was ~48.2 μg/m^3^. For April, the average in 2019 was 56.6 μg/m^3^, while in 2020, the average was ~42.16 μg/m^3^ (Figure 1).

Notable reductions in the average NO_2_ concentrations at single traffic stations in March and April 2020 compared to those in the previous year are evident. In particular, the statistical analysis showed significant differences in the NO_2_ concentration in March 2019/2020 for the Bari-Cavour monitoring station (*p* < 0.001) and in April 2019/2020 for the Bari-Caldarola (*p* = 0.033) and Bari-Cavour stations (*p* < 0.001) (Table 1).

**Table 1:** NO_2_ March-April 2019/2020 concentrations.

**NO_**2**_**	**Bari-Caldarola station**				**Bari-Cavour station**				**Bari-CUS station**			
	**μg/m**^****3****^				**μg/m**^****3****^				**μg/m**^****3****^			
	**Average**	**Min-max**	**SD**	***p*-value**	**Average**	**Min-max**	**SD**	***p*-value**	**Average**	**Min-max**	**SD**	***p*-value**
March 2019	65.9	50–94	12.87	0.569	72.3	43–117	19.37	<0.001	48.5	18–84	17.82	0.465
March 2020	59.1	23–84	18.17		45.2	12–75	17.47		40.3	4–88	24.38	
April 2019	63.4	39–89	10.71	0.033	72.3	47–113	15.45	<0.001	34.0	7–56	13.43	0.071
April 2020	49.2	13–114	34.21		38.3	18–73	15.20		38.0	5–99	22.63	

**Figure 2:**
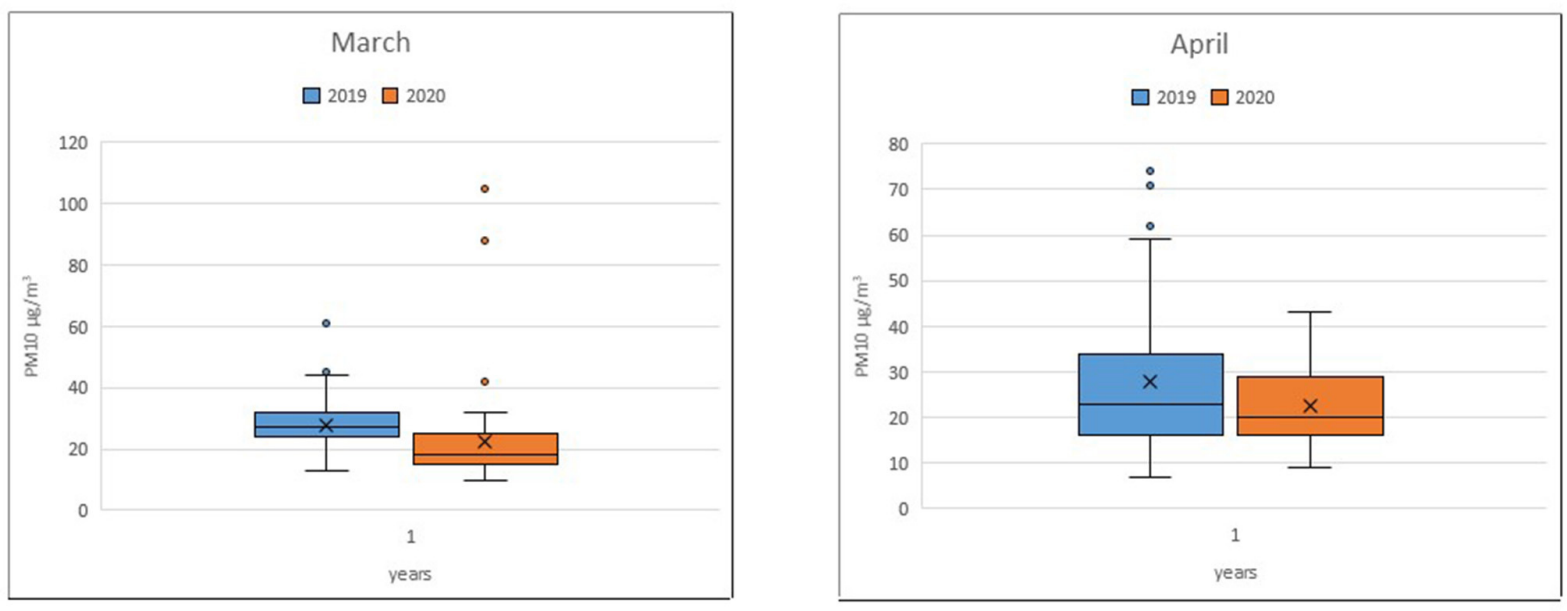
Traffic station PM10 monthly average.

Regarding PM10 levels, the average in March 2019 was ~26.9 μg/m^3^, while that in March 2020 was ~22.9 μg/m^3^. For April, the average concentration of PM10 in 2019 was 27.9 μg/m^3^, while in 2020, the average was ~22.4 μg/m^3^ (Figure 2).

The average concentrations of PM10 at the individual traffic stations in the months of March and April 2020 showed no particular variation compared to those in the same months of the previous year, except for Bari-Caldarola Station in March 2019/2020 (*p*-value < 0.001) and in April 2019/2020 (*p*-value = 0.04) (Table 2). This result is in contrast with the clear reduction in emissions from vehicular traffic during the lockdown phase and is likely attributable to the influence of weather conditions on particulate concentrations.

**Table 2:** PM10 March-April 2019/2020 concentrations.

**PM10**	**Bari-Caldarola station**				**Bari-Cavour station**				**Bari-Cus station**			
	**μg/m**^****3****^				**μg/m**^****3****^				**μg/m**^****3****^			
	**Average**	**Min-max**	**SD**	***p*-value**	**Average**	**Min-max**	**SD**	***p*-value**	**Average**	**Min-max**	**SD**	***p*-value**
March 2019	30.1	13–61	9.44	<0.001	25.7	14–38	5.62	0.138	25.0	10–38	6.78	0.374
March 2020	18.4	10–31	5.68		25.9	11–105	20.98		24.4	8–104	21.45	
April 2019	29.9	9–71	16.93	0.04	27.6	11–72	14.80	0.596	26.2	7–24	16.23	0.75
April 2020	20.6	9–35	7.58		23.7	10–41	8.89		22.8	9–43	9.16	

## Discussion and Conclusion

This is the first assessment of the effects of restrictive measures in the Apulia region on air pollution during the national lockdown period in Italy. These adopted mobility measures have seriously weakened the national socioeconomic balance; however, the significant impact on travel and the consequent reduction in pollutant emissions from transportation has had a positive effect on air quality.

Vehicular transportation is the sector that experienced the greatest impact due to the drastic and significant decline in mobility during the period of restriction. The transportation sector has a major impact on the national emissions budget. Pollutants, such as atmospheric particulate matter (PM10, PM2.5) and nitrogen dioxide (NO_2_), whose levels are closely related to patterns of vehicular traffic, generally reach high concentrations in urban areas. In the latest “Italian Emission Inventory 1990–2018” report, The Higher Institute for Environmental Protection and Research (ISPRA) notes that this sector is responsible for 43.5% of the total emissions of nitrogen oxides, 19.9% of carbon monoxide, 11.9% of NMVOCs (Non-methane Volatile Organic Compounds) and 11.8 and 10.5% of total PM10 and PM2.5, respectively (30). At the regional level, according to the latest “Regional Inventory of Emissions into the Atmosphere - INEMAR Puglia,” road transportation is the main emission source of nitrogen oxides, accounting for 42.5% of total annual emissions. Regarding dust, road transportation is responsible for 18.4 and 18.5% of PM10 and PM2.5 total emissions/year, respectively (31).

In the Apulian region, data collected by the monitoring stations maintained by the Regional Environmental Protection Agencies (ARPA) in March–April 2020 showed that the daily concentrations of air pollutants such as NO_2_ (a primary pollutant directly produced by vehicular emissions) were reduced at all the selected monitoring stations. In particular, in the municipal area of Bari, the statistical analysis showed significant differences in the average NO_2_ concentration in March 2019/2020 for the Bari-Cavour monitoring station (*p* < 0.001) and in April 2019/2020 for the Bari-Caldarola (*p* = 0.033) and Bari-Cavour stations (*p* < 0.001). On the contrary the monthly average PM10 concentration showed no significant difference between March–April 2019 and the same months in 2020 except for Bari-Caldarola station^6^. Different authors have reported improvements in air quality after the lockdown period in several countries, especially in places that had relatively high levels of air pollutants before the pandemic period (for example, Brazil, China, and India) (32). In accordance with our data, a Spanish study observed that after 2 weeks of lockdown in the city of Barcelona, NO_2_ was reduced by half, while there was a lower reduction in the PM10 concentration (33). The same result was reported by Otmani et al. in a study conducted in Salé City (Morocco); PM10, NO_2_, and SO_2_ concentrations were reduced by more than half during the COVID-19 lockdown period, but the most significant variation was observed for NO_2_ (34). Mahata et al. obtained different results in a study on air quality of the megacity Delhi; the study evaluated seven pollutants (PM10, PM2.5, SO_2_, NO_2_, CO, O_3_, and NH_3_) at 34 monitoring stations spread throughout the megacity. Among the measured pollutants, PM10 and PM2.5 concentrations were reduced by approximately half compared to those during the pre-lockdown period, while there was a lower reduction in other pollutants, such as NO_2_, CO, and NH_3_ (35). In contrast to other studies demonstrating that air quality improved during the COVID-19 pandemic, air quality research conducted in New York City revealed no significant change in air quality compared to that during the same periods in 2015–2019. The different results obtained in that study may be explained by the fact that New York City has lower baseline concentrations of air pollutants than other countries that were studied (36).

The preliminary data suggest that the epidemiological emergency attributable to SARS-CoV-2 has radically changed citizen mobility in Italy, particularly affecting the transportation of freight and persons and the free movement of individuals. However, this historical event, albeit dramatic in some respects, has resulted in a significant improvement in air quality.

However, there are still questions about the proportion of the abatement of pollution directly related to the lockdown without meteorological interference, and why PM10 levels were not reduced as much as NO_2_ levels. Meteorological conditions have an important influence on the formation of air pollution and changes in pollutant concentration. Among these, temperature and wind speed are generally considered to be two major factors affecting the concentration of air pollutants. (37). Based on the real-time data of several air pollutant concentrations (PM2.5, PM10, CO, SO_2_, NO_2_, and O_3_) and daily meteorological data from June 2014 to February 2019, a recent study by Yansui Liu investigates the spatio-temporal characteristics of air pollutant concentration and meteorological factors in China. Except for O_3_, the concentration of other air pollutants at most sites was significantly and negatively correlated with average wind speed, precipitation and relative humidity, but positively correlated with atmospheric pressure. Moreover, the degree of impact depended on the type of pollutants and the geographical location of the stations. In the North Plain, temperature was recognized as the dominant factor affecting PM10 concentration, while wind speed and relative humidity as the dominant factors in Northeast. Relative humidity was identified as the dominant factor for PM10 concentration changes at most sites. For NO_2_, in addition to temperature, atmospheric pressure has also been identified as one of the leading meteorological factors for changes in pollutant concentration in the eastern coastal and northeastern regions. Relative humidity also had a significant impact on NO_2_ concentration in the Beijing-Tianjin-Hebei region (38). These results are consistent with previous studies (39, 40). The role of meteorological variables is not quantified: this is a limitation of our study. PM10 concentration levels in urban environment are also influenced by long range transport form remote source areas. In this regard on March 30, 2020, despite restrictive measures, Bari-Cavour and Bari CUS traffic stations recorded levels exceeding the daily legal limit for PM10 (105 and 104 μg/m^3^). This was probably connected to a rare advection of non-anthropogenic fine dust, carried by current from the East. Modeling simulations, performed by ARPA agencies as part of the new SNPA-ASI Copernicus platform, showed that it was dust from the Asian desert, bordering the Caspian Sea. The natural origin of PM is further demonstrated by the fact that in the last days of March 2020 there was a very significant increase in PM10, but not in PM2.5, suggesting that the dust of these days are coarse, compatible with a terrigenous origin ^6^.

In Italy, the legislative and technological measures implemented in recent years have produced environmental benefits, such as the introduction of new air quality standards, regulation of the use of fuels, and establishment of a number of monitoring stations for pollutants. However, the need to maintain restrictive measures during the post-emergence phase could affect the current sustainable mobility policies. Interpersonal distancing and compliance with hygiene standards could encourage the use of private vehicles, discourage the use of public transportation and compromise all progress on sustainable mobility.

Therefore, it would be desirable to implement the following strategies ^7^:

- Redistribute the demand for mobility, for example, by expanding and diversifying the start times of workplaces, public offices, schools, and shopping centers;
- Reduce the demand for mobility, for example, by encouraging teleworking;
- Promote shared mobility solutions, such as bike sharing, scooter sharing and electric micromobility;
- Encourage the building of pedestrian and cycling infrastructure, such as bike lines;
- Redesign urban spaces according to proximity criteria. In this regard, the French urban regeneration project called “*La ville du quart d'heure”* is exemplary. In this project, each district of the city will be provided a number of useful services for everyday life (e.g., grocery stores, green areas, healthcare facilities) that are accessible by foot or by bicycle with a maximum 15-min travel time.

## Data Availability Statement

The original contributions presented in the study are included in the article/supplementary material, further inquiries can be directed to the corresponding author/s.

## Author Contributions

LV: project administration. ST, NM, CL, and LV: conceptualization, supervision, and project administration. LD, AC, and EC: writing—original draft preparation, review, and editing. SS and MD: data curation. AP: formal analysis. VB and LA: investigation. All authors have read and agreed to the published version of the manuscript.

## Conflict of Interest

The authors declare that the research was conducted in the absence of any commercial or financial relationships that could be construed as a potential conflict of interest.

## Footnotes

^1^http://www.protezionecivile.gov.it/documents/20182/1227694/Summary+of+measures+taken+against+the+spread+of+C-19/c16459ad-4e52-4e90-90f3-c6a2b30c17eb

^2^https://www.esa.int/Applications/Observing_the_Earth/Copernicus/Sentinel-5P/Coronavirus_lockdown_leading_to_drop_in_pollution_across_Europe

^3^https://osservatoriocpi.unicatt.it/cpi-Lockdown%20e%20mobilit%C3%A0.pdf

^4^https://www.isfort.it/wp-content/uploads/2020/06/200521_Isfort_MobilitaItaliani_Lockdown_DEF.pdf

^5^https://lab.octotelematics.com/

^6^https://www.snpambiente.it/2020/04/08/qualita-dellaria-in-puglia-in-concomitanza-dellemergenza-covid-19/

^7^https://www.kyotoclub.org/medialibrary/Rapporto_Mobilitaria2020_DEFINITIVO_maggio2020.pdf
